# A brief history and popularity of methods and tools used to estimate micro‐evolutionary forces

**DOI:** 10.1002/ece3.8076

**Published:** 2021-09-16

**Authors:** Jonathan Kidner, Panagiotis Theodorou, Jan O. Engler, Martin Taubert, Martin Husemann

**Affiliations:** ^1^ General Zoology Institute for Biology Martin Luther University Halle‐Wittenberg Halle (Saale) Germany; ^2^ Terrestrial Ecology Unit Department of Biology Ghent University Ghent Belgium; ^3^ Aquatic Geomicrobiology Institute for Biodiversity Friedrich Schiller University Jena Jena Germany; ^4^ Centrum für Naturkunde University of Hamburg Hamburg Germany

**Keywords:** drift, migration, mutation, population genetics, selection, software, user bias

## Abstract

Population genetics is a field of research that predates the current generations of sequencing technology. Those approaches, that were established before massively parallel sequencing methods, have been adapted to these new marker systems (in some cases involving the development of new methods) that allow genome‐wide estimates of the four major micro‐evolutionary forces—mutation, gene flow, genetic drift, and selection. Nevertheless, classic population genetic markers are still commonly used and a plethora of analysis methods and programs is available for these and high‐throughput sequencing (HTS) data. These methods employ various and diverse theoretical and statistical frameworks, to varying degrees of success, to estimate similar evolutionary parameters making it difficult to get a concise overview across the available approaches. Presently, reviews on this topic generally focus on a particular class of methods to estimate one or two evolutionary parameters. Here, we provide a brief history of methods and a comprehensive list of available programs for estimating micro‐evolutionary forces. We furthermore analyzed their usage within the research community based on popularity (citation bias) and discuss the implications of this bias for the software community. We found that a few programs received the majority of citations, with program success being independent of both the parameters estimated and the computing platform. The only deviation from a model of exponential growth in the number of citations was found for the presence of a graphical user interface (GUI). Interestingly, no relationship was found for the impact factor of the journals, when the tools were published, suggesting accessibility might be more important than visibility.

## Graphical abstract

Multiple booms in software analyzing population genetics have occurred with changing technology/techniques. Both established and novel techniques have been developed providing a plethora of different analysis methods and programs for the researcher. We aim to provide an overview for the major micro‐evolutionary forces over these methods, providing a resource for researchers to assess the diversity of software in the field.

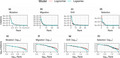

## 1 INTRODUCTION

The modern synthesis (Mayr & Provine, 1980) has revolutionized our perception of micro‐evolution by providing a conceptual and theoretical framework to investigate its processes. In this synthesis, four main forces driving evolution were described as contributing to changes in allele frequencies: mutation (*μ*), gene flow (m), drift (estimated as its inverse effective population size (N*_E_*)), and selection (s). The modern synthesis provides the theoretical framework to predict the effects of each of these forces in various settings. A chronology is apparent with many of the tools (particularly the older programs) still implementing algorithms developed earlier during the modern synthesis, alongside more recently developed ones adopted for different marker systems.

The types of population genetic markers have broadened substantially from its initial emergence; the earlier studies used allozymes to estimate population differentiation (for a review, see Allendorf, 2016). These were largely replaced by genetic markers, (Sunnucks, 2000), such as random primer binding (RAPD), restriction site polymorphisms (RFLP, AFLP), fragment length variation of satellite DNA (e.g., microsatellites), or sequence polymorphisms (single nucleotide polymorphisms, SNPs). Two main categories of molecular markers can be defined: dominant markers, absent of the ability to determine homo‐ or heterozygosity (AFLP, RFLP); and codominant, that can be used to determine homo‐ or heterozygosity (microsatellites, allozymes, nucleotide variation). Codominant markers have consequently received increasing attention among researchers due to their greater information content.

While analyzing a specific allozyme, or microsatellite would provide a single locus in a study, the development of mass sequencing and SNP‐typing has enabled the creation of massive datasets involving thousands to millions of loci. However, the use of high‐throughput sequencing (HTS) techniques introduced a whole new range of analytical problems for researchers studying genetic diversity, while increasing the scope for testing the predictions of the modern synthesis (Davey & Blaxter, 2010). This has required the development of new techniques to incorporate the issues introduced through mass sequencing, reducing false positives, while maintaining sensitivity (Cavalli‐Sforza, 1998). These new methods now populate the software tools for estimating population genetic parameters alongside techniques that are developed for single loci. This requires the researchers using these software suites to be able to discern their needs, and the provisions of the individual programs/tools.

The first population genetic programs were based on goodness of fit tests (chi‐square, or F‐based tests: Allendorf, 2016; Excoffier & Heckel, 2006; Labate, 2000). However, these approaches became increasingly intractable as the null models (and the understanding of the complexity of the data) increased in complexity. Approaches such as maximum likelihood (ML) were developed as an alternative, while allowing for the efficient exploration of more complicated multi‐dimensional parameter space. Later on, the “Bayesian Revolution” led to further developments in genetic data analyses that allowed for even more complex models in inferring the distributions of estimates of micro‐evolutionary forces (Beaumont & Rannala, 2004); as well as avoiding some critiques leveled at the ML approaches (Stigler, 2007). The subsequent use of computational techniques to simulate parameter distributions helped to reduce the computational demand involved in Bayesian testing, leading to the development of approximate Bayesian computation (ABC, Csillery et al., 2012) among others.

All of these means of inference are maintained within the current community of software and tools to analyze population genetics, and multiple comparisons of the tools and algorithms have been performed (Excoffier & Heckel, 2006; Labate, 2000; Putman & Carbone, 2014). However, a comprehensive overview of population genetic software (including more recent publications) and an analysis of their usage is currently missing.

Here, we provide a short overview of the methods, programs, and resources used to estimate mutation, gene flow, drift (in terms of N_E_), and selection. We furthermore provide an extensive list of approximately 100 programs for population genetic analyses, and briefly discuss their functionalities and differences. On these programs, we investigate popularity and usage patterns within the scientific community, our null model being that the probability of finding a new citation for a particular program is dependent on the previous total number of citations observed (of the program, Taylor, 1961). This leads to citation distributions described by log‐normal, and series models, which can be more formally described through Taylor's power law mean‐variance relationships. Subsequently, we also investigate the relationship between niche breadth and range size of a program. We use niche to refer to how research developments lead to new opportunities to expand research areas, for example, the development of HTS, or machine learning; range is used to describe the occupation of different niches by individual programs. These results are discussed in terms of program complexity, user friendliness, year of publication, impact factor (IF) of the journal at the time of publication, computing platform, and the type and number of population genetic parameters estimated.

## 2 METHODS

We generated an extensive list of population genetic software by using a combination of Google searches, Google Scholar, Web of Knowledge, and the bioinformatic search engine omictools.com. For each program, we recorded the evolutionary force(s) or proxy of force(s) it is able to estimate, the operating system it runs on, the year of publication, presence–absence of a graphical user interface (GUI), and the journal's IF at the time of publication. Further, we downloaded citation records for all articles from ISI web of knowledge (25.02.2019), which were used as a proxy for citation bias. In total, we report 101 programs and scripts. For 96 of these, where papers were available, we downloaded citation records. From these 96, we only used those published before 2014 (to reduce the impact of recently emerged tools on our analysis), which resulted in 80 papers (software packages) included in our analyses. Analysis of citation records was performed in R v. 3.6.3 (R Core Team, 2018) using customized versions of the scripts published by Keil et al. (2018). These are provided in the Appendix S1. The analyses included the use of the R packages: *plyr* (Wickham, 2011), *ggplot2* (Wickham, 2016), *reshape2* (Wickham, 2007), and *MASS* (Venables & Ripley, 2002).

In our analyses, we first investigated the programs using species abundance distributions (SAD*s*); these were analyzed with log‐normal and log‐series models being fitted to citation data collected from ISI web of knowledge. Assuming that each use and citation of the software occurs independently, these models provide a good description of the abundance in the citation records (Baldridge et al., 2016). We used these two models as they are simple descriptors of SADs, the log‐series fitting better when more singletons (software with single citations) are observed in the dataset, compared to the log‐normal. The log‐series model was implemented with the *sads* package in R, while the logged mean and variance were used for the log‐normal model. An identical approach to sampling as in Keil et al. (2018) was done, with the average of 500 model runs taken for generating the expected values. For Taylor's power law, we adopted the number of times the software was cited as its citation bias, according to its publication record on ISI web of knowledge. The citation bias in our data was tested for deviations from a purely random model of citation growth with a linear model on log‐transformed means and variances. This model describes how the process of accumulating new citations is based on the exponential growth of the number of citations (Keil et al., 2018; Taylor, 1961). A process that can be described by a chaotic, random walk that holds true when the mean–variance relationship is approximately equal to 2. When the mean–variance relationship is less than 2 additional parameters are required in the growth model. Further, we tested the effects of operating system (Microsoft Windows, Linux, macOS), parameter (mutation, drift, migration, selection), and GUI on citation bias using nonparametric Kruskal–Wallis tests implemented in R. For testing the effect of the IF of the journal, we used the robust regression model from the *MASS* package (Venables & Ripley, 2002). All programs included in the analyses are listed in Table 1 together with their references; download links and DOIs are provided in the Appendix S1, where available.

**TABLE 1:** List of population genetics software

Program name	Year	*μ*	m	N*_E_*	s	Windows	Mac	Linux	IF	GUI	Reference
2mod	1999			Yes		Yes			NA	NA	Ciofi et al. (1999)
abc	2012		Yes	Yes		Yes	Yes	Yes	5.924	No	Csillery et al. (2012)
ABCtoolbox	2010		Yes	Yes			Yes	Yes	3.029	No	Wegmann et al. (2010)
adegenet	2008		Yes			Yes	Yes	Yes	4.328	No	Jombart (2008)
AgeStructure	2010			Yes		Yes			5.659	NA	Wang et al. (2010)
ANGSD	2013				Yes			Yes	2.672	No	Korneliussen et al. (2014)
Arlequin3	2005		Yes		Yes	Yes			NA	Yes	Excoffier and Schneider (2005)
Arlequin35	2010		Yes	Yes	Yes	Yes			1.631	Yes	Excoffier and Lischer (2010)
Ballet	2014			Yes	Yes			Yes	7.528	No	DeGiorgio et al. (2014)
Bayenv	2010				Yes		Yes	Yes	4.866	No	Gunther and Coop (2013)
BayesAss	2003		Yes			Yes	Yes	Yes	4.276	No	Wilson and Rannala (2003)
Bayescan	2012				Yes	Yes	Yes	Yes	4.002	Yes	Foll and Gaggioti (2008)
BayesFST	2004				Yes	Yes	Yes	Yes	2.261	No	Balding (2003)
BayPass	2015				Yes	Yes	Yes	Yes	4.644	No	Gautier (2015)
BEAST	2007	Yes		Yes		Yes	Yes	Yes	4.091	Yes	Drummond and Rambaut (2007)
BEAST17	2012	Yes		Yes		Yes	Yes	Yes	10.353	Yes	Drummond et al. (2012)
BEAST2	2014	Yes		Yes		Yes	Yes	Yes	NA	Yes	Bouckaert et al. (2014)
BIMr	2008		Yes			Yes	Yes	Yes	4.002	Yes	Faubet and Gaggiotti (2008)
bz_rates	2015	Yes				Yes	Yes	Yes	2.91	Yes	Gillet‐Markowska et al. (2015)
Colony	2009			Yes		Yes	Yes	Yes	1.259	NA	Jones and Wang (2009)
CoNe	2004			Yes		Yes			4.289	NA	Anderson (2005)
DetSel	2013				Yes	Yes	Yes	Yes	1.707	No	Vitalis et al. (2003)
diCal	2013			Yes		Yes	Yes	Yes	4.866	NA	Sheehan et al. (2013)
DIYABC	2008		Yes	Yes		Yes	Yes	Yes	4.328	NA	Cornuet et al. (2008)
DIYABC2	2014		Yes	Yes		Yes	Yes	Yes	4.981	Yes	Cornuet et al. (2014)
dlik	1998			Yes		Yes			NA	NA	O'Ryan et al. (1998)
DnaSP	2009				Yes	Yes			4.926	Yes	Librado and Rozas (2009)
EggLib	2014				Yes	Yes	Yes	Yes	2.808	No	De Mita and Siol (2012)
EigenGWAS	2015				Yes	Yes	Yes	Yes	3.801	No	Chen et al. (2015)
Estim	2001		Yes	Yes		Yes			NA	NA	Vitalis and Couvet (2001)
FALCOR	2009	Yes				Yes	Yes	Yes	4.926	Yes	Hall et al. (2009)
FLK	2010				Yes	Yes	Yes	Yes	4.087	No	Bonhomme et al. (2010)
Garcia_script	1998	Yes				Yes			0.863	No	Garcia‐Dorado and Marin (1998)
GenAlEx	2006		Yes			Yes	Yes	Yes	4.894	Yes	Peakall and Smouse (2006, 2012)
Geneland	2005		Yes			Yes	Yes	Yes	1.219	No	Guillot et al. (2005)
Genetree	2000	Yes	Yes			Yes		Yes	1.833	NA	Bahlo and Griffiths (2000)
Gimlet	2002			Yes		Yes			NA	Yes	Valière (2002)
GONe	2012			Yes		Yes			7.432	Yes	Coombs et al. (2012)
HacDivSel	2017				Yes		Yes	Yes	2.766	No	Carvajal‐Rodriguez (2017)
hapbin	2015				Yes			Yes	13.649	No	Maclean et al. (2015)
hapflk	2013				Yes		Yes	Yes	4.866	No	Fariello et al. (2013)
IM	2001	Yes	Yes	Yes		Yes	Yes	Yes	4.803	NA	Nielsen and Wakeley (2001)
Ima	2007	Yes	Yes	Yes		Yes	Yes	Yes	9.598	No	Hey and Nielsen (2007)
IMa2	2010	Yes	Yes	Yes		Yes	Yes	Yes	5.51	No	Hey (2010)
Ima2p	2015	Yes	Yes	Yes		Yes	Yes	Yes	5.298	No	Sethuraman and Hey (2016)
Keightly_script	1998	Yes				Yes	Yes	Yes	4.45	No	Keightley (1998)
Lamarc	1999		Yes	Yes		Yes	Yes		4.221	No	Beerli and Felsenstein (1999)
Lamarc2	2006	Yes	Yes	Yes		Yes	Yes	Yes	4.894	NA	Kuhner (2006)
LDNe	2008			Yes		Yes	Yes	Yes	NA	Yes	Waples and Do (2008)
Lositan	2008				Yes	Yes	Yes		3.781	NA	Antao et al. (2008)
McDonald‐Kreitman_online	2008				Yes	Yes	Yes	Yes	6.878	Yes	Egea et al. (2008)
MCLEEPS	2000			Yes			Yes		4.687	No	Anderson et al. (2000)
mdiv	2001		Yes			Yes			4.803	No	Nielsen and Wakeley (2001)
MEAdmix	2006			Yes		Yes			4.242	No	Wang (2006)
MEGA6	2013				Yes	Yes	Yes	Yes	14.308	Yes	Tamura et al. (2013)
MigEst	2014		Yes			Yes			6.494	NA	Wang (2014)
MIGRATE_N	2013	Yes	Yes			Yes	Yes	Yes	4.087	No	Beerli and Palczewski (2010)
MIMAR	2007		Yes	Yes					5.169	No	Becquet and Przeworski (2007)
MISAT	1997			Yes		Yes			4.275	No	Nielsen (1997)
MLNe	2001		Yes	Yes		Yes			2.317	NA	Wang (2001)
mlRho	2010	Yes					Yes	Yes	6.457	No	Haubold et al. (2010)
MSMC	2014			Yes			Yes	Yes	29.352	No	Schiffels and Durbin (2014)
Nb_HetEx	2008			Yes		Yes			1.775	NA	Zhdanova and Pudovkin (2008)
NeEstimator	2004			Yes		Yes	Yes	Yes	NA	Yes	Peel et al. (2004)
NeEstimator2	2014			Yes		Yes	Yes	Yes	3.712	Yes	Do et al. (2014)
neighbor	2002		Yes			Yes			3.014	NA	Burczyk et al. (2002)
NM+	2010		Yes			Yes			1.631	NA	Chybicki and Burczyk (2010)
nSl	2014				Yes		Yes		9.105	No	Ferrer‐Admetlla et al. (2014)
Omegamap	2006				Yes	Yes	Yes		4.242	No	Wilson and McVean (2006)
OmegaPlus	2010				Yes	Yes		Yes	5.323	No	Alachiotis et al. (2012)
ONeSamp	2008			Yes		Yes	Yes	Yes	NA	No	Tallmon et al. (2008)
OutFLANK	2015				Yes	Yes	Yes	Yes	3.148	No	Whitlock and Lotterhos (2015)
pcadapt	2017				Yes	Yes	Yes	Yes	7.059	No	Luu et al. (2017)
pegas	2010		Yes			Yes	Yes	Yes	4.877	No	Paradis (2010)
Pool_hmm	2012				Yes	Yes	Yes	Yes	10.353	No	Boitard et al. (2013)
PopABC	2009	Yes	Yes	Yes		Yes	Yes	Yes	4.926	No	Lopes et al. (2009)
popgene	1997				Yes	Yes			0.231	Yes	Yeh and Boyle (1997)
PSMC	2011			Yes				Yes	36.28	No	Li and Durbin (2011)
rehh	2012				Yes	Yes	Yes	Yes	5.468	No	Gautier and Vitalis (2012)
rehh2	2017				Yes	Yes	Yes	Yes	7.059	No	Gautier et al. (2017)
RpackageNB	2015			Yes		Yes	Yes	Yes	4.644	No	Hui and Burt (2015)
SAMOVA	2002		Yes			Yes			3.014	NA	Dupanloup et al. (2002)
SelectionHapStats	2018				Yes			Yes	3.564	No	Harris et al. (2018)
Selecton	2007				Yes	Yes	Yes	Yes	6.954	No	Stern et al. (2007)
SelEstim	2014				Yes	Yes	Yes	Yes	5.963	No	Vitalis et al. (2014)
selscan	2014				Yes	Yes	Yes	Yes	9.105	No	Szpiech and Hernandez (2014)
SNeP	2015			Yes		Yes	Yes	Yes	NA	No	Barbato et al. (2015)
SPAM	2004		Yes			Yes			1.511	Yes	Debevec et al. (2000)
spatpg	2016			Yes	Yes			Yes	6.086	No	Gompert (2016)
Structure	2000		Yes			Yes	Yes	Yes	4.687	Yes	Pritchard et al. (2000)
SweeD	2013				Yes		Yes	Yes	14.308	No	Pavlidis et al. (2013)
Sweep	2005				Yes	Yes	Yes	Yes	30.432		Sabeti et al. (2002)
sweepfinder	2006				Yes			Yes	10.139	No	Nielsen et al. (2005)
SweepFinder2	2016				Yes		Yes	Yes	7.307		DeGiorgio et al. (2016)
TempoFs	2007			Yes		Yes		Yes	4.001	No	Jorde and Ryman (2007)
Tess	2007		Yes			Yes	Yes	Yes	1.257	No	Chen et al. (2007)
ThetaCurve	2012	Yes				Yes	Yes	Yes	NA	No	n.a.
tm3	2002			Yes		Yes			4.483	NA	Berthier et al. (2002)
tmvp	2003			Yes		Yes			4.276	NA	Beaumont (2003)
TreeSelect	2011				Yes	Yes	Yes	Yes	10.603	No	Bhatia et al. (2011)
WFABC	2015		Yes	Yes	Yes	Yes	Yes	Yes	5.298	No	Foll et al. (2015)

Note

101 Programs for predicting population genetic parameters. Each software/Program is described by the types of parameters it estimates (mutation—*µ*, migration—m, effective population size (drift)—N*_E_*, or selection—s), as well as the platform it is available on (Windows, Mac, or Linux) and the reference and date of publication. Links to the programs are provided in the Appendix S1.

## 3 RESULTS AND DISCUSSION

### 3.1 Estimating the rate and effect of mutation

Mutations (i.e., single nucleotide mutations; insertions/deletions and chromosome rearrangements) are the ultimate source of genetic variation, and the precise estimation of mutation rates (*μ*) is important to understand the mechanisms of evolution (Lynch, 2010). However, estimating mutation rates remains relatively difficult (Kondrashov & Kondrashov, 2010), mainly due to the randomness of the process and the generally low mutation rates observed in eukaryotes.

The most direct techniques to estimate mutation rates, applicable only to microorganisms or model species with short generation times, are fluctuation tests in bacteria and yeast, and mutation accumulation experiments in nonmammalian model systems (Foster, 2006; Luria & Delbrück, 1943; Lynch et al., 2008; Rosche & Foster, 2000). In fluctuation experiments, a low number of wild‐type cells are used to inoculate large numbers of parallel cultures under nonselective conditions; these are then moved to selective media to identify mutants. Even though the fluctuation experiment is conceptually simple, the mathematics to estimate the rate of mutation from the frequency of mutants remain challenging (Zheng, 2017). The most widely used statistical method to estimate μ from fluctuation experiments is the Lea–Coulson method of the median (LC; Lea & Coulson, 1949). However, more sophisticated statistical analyses have emerged that can estimate mutation rates from fluctuation experiments more accurately, incorporating several statistical estimators. The MSS‐MLE method implemented in the web tool FALCOR uses an initial estimate of μ to generate the probability of observing n mutants on a selective medium and uses the complete dataset from a fluctuation experiment, rather than just summary statistics, providing more statistical power (Hall et al., 2009). In comparison, the web tool bz‐rates employs a generating function estimator, allowing the calculation of μ while controlling for differential growth rates (Gillet‐Markowska et al., 2015). As an alternative, the R package rsalvador (Zheng, 2017) provides various methods for computing ML estimates of μ with likelihood ratio‐based confidence intervals.

Mutation accumulation experiments (MA) represent another method to directly estimate mutation rates (Luria & Delbrück, 1943; Lynch et al., 2008). In such experiments, isogenic lines of model organisms randomly accumulate mutations through several generations of inbreeding. The resulting loss of fitness (e.g., in terms of growth rate and reproductive success) compared to control lines (ΔM) and the fitness variance (ΔV) among the lines is then used to infer mutation rates, for example, using Bateman–Mukai (BM, Bateman, 1959; Mukai, 1964), ML (Wloch et al., 2001), or minimum distance methods (MD, Garcia‐Dorado, 1997). The performance of BM, ML, and MD methods has been assessed using simulation datasets and concluded that MD methods produce estimates with the lowest bias and sampling variance (Garcia‐Dorado & Gallego, 2003). A more thorough review on the performance of the above methods can be found in Garcia‐Dorado and Gallego (2003).

The majority of tools, for estimating mutation rate, applicable to a taxonomically wider range of organisms are based on genetic data. These methods can compare a variety of neutral homologous sequences from related species with calibrated divergence times to infer mutation rates, based on the simple assumption that the rate of neutral sequence divergence is equal to the mutation rate (Kimura, 1968). For the user, the choice of appropriate neutral markers and availability of appropriate sister species for comparison are among the biggest challenges involved in using these methods (Kondrashov & Kondrashov, 2010). Many software suites can be used to estimate divergence rates and hence to infer mutation rates indirectly. One of such programs is beast (Bouckaert et al., 2014), a software that uses sampling across phylogenetic tree space to infer a variety of population parameters; the locus specific mutation rate being one of which.

The development of HTS technologies helped to minimize the limitations of both the direct (e.g., fitness‐based assays) and indirect methods (e.g., small number of loci) and provided novel means to estimate mutation rates. The application of HTS technologies in fluctuation and mutation accumulation experiments has allowed a direct estimate of mutation rates irrespective of phenotypic or fitness effects (Katju & Bergthorsson, 2019; Nishant et al., 2009). In addition, long‐term mutation accumulation experiments are no longer required; data on few generations suffices, expanding the possibility to obtain accurate measures of mutations in nonmodel species. Furthermore, the development of HTS technologies allowed the detection of germline mutation rates by comparing genomic sequences between subsequent generations, as well as estimating mutation rates by sequencing individuals of pedigreed families (e.g., parent–offspring). Pedigree sequencing is a very promising method applicable to any organism; however, such method requires a large number of individuals (Keightley et al., 2014) and special care should be taken during the analysis to avoid false variant calls and the risk for increased false‐positive rates.

### 3.2 Estimation of gene flow

Migration, also a creative force introducing new variants into a population, is synonymous with gene flow in population genetics and refers to the spatial movement of alleles (Broquet & Petit, 2009). The parameter estimated can either be the effective number of migrants (N_E_M_E_), or the effective migration rate (M_E_). N_E_M_E_ estimates the migration rate in terms of the effective population size of migrants, while the M_E_ estimates the net migration rate per generation (Waples & Gaggiotti, 2006). Both parameters can be estimated based on F_ST_ (Wright, 1931, 1940), yet doing so generally entails issues due to the presence of confounding factors (Cayuela et al., 2018; Whitlock & McCauley, 1999) of F_ST_ estimation. A brief flavor of which can be described through F_ST_ also being the product of drift (1/N_E_) and hence consequently being affected by all factors contributing to N_E_, including demographic and adaptive processes. With many alternative tools existing, the determination of the most appropriate method for analyzing gene flow will generally depend on the type and amount of available data to inform the models.

In a general approach to investigating hypotheses of migration, an initial step is to assign samples to a population. Thereby generating a population structure within which the migration rates can be estimated. Multiple approaches exist to do so, one of which is the simulation style approach of Markov Chain Monte Carlo as used in bayesass (Wilson & Rannala, 2003), a second is the likelihood approach as in migest/popcluster (Wang, 2014). While these tools produce estimates of population structure to estimate gene flow, the tools structure and spam provide more detailed estimates and measures of population structure in samples (Debevec et al., 2000; Pritchard et al., 2000). spam provides the ability to identify the contribution of multiple populations to a single sample (it was originally developed for use with fisheries data; Debevec et al., 2000). In contrast, structure is most frequently used to identify stratification and (sub‐) divisions within populations, providing sample assignment probabilities (Pritchard et al., 2000). Unfortunately, structure performs less well with more continuously defined populations; in these scenarios, samova can provide an alternative approach, apportioning genetic variance maximally between groups of populations (Dupanloup et al., 2002).

Many software suites offer opportunities to investigate population structure further, including the provision of more detailed statistics and many more graphical representations surrounding population assignment. Similarly to spam and structure—genalex, tess3, and geneland (Chen et al., 2007; Guillot et al., 2005; Peakall & Smouse, 2006)—provide statistics on individual assignment probabilities and distributions. genalex offers many different types of analysis and graphical representations, providing a high level of detail on the population assignment statistics, as well as the allelic breakdown over multiple spatial dimensions. This is similar to tess3 and geneland. However, these programs use different techniques for inferring the individual assignment probabilities and provide different summary statistics: the ancestry coefficients produced by tess3 additionally allow for investigations of neutrality among the available loci, when population substructuring is strong. Both geneland and tess3 offer plotting features, that integrate maps and assignment probabilities to provide a fully geographical analysis of populations.

The assignment approaches mentioned in the previous paragraphs are also used in bayesass, migest/popcluster, and arlequin (Excoffier & Schneider, 2005; Excoffier & Lischer, 2010; Wang, 2014; Wilson & Rannala, 2003, respectively). However, these assignment approaches are used to produce estimates for rates of recent migration, providing a valuable tool for ecology and conservation research, but may not reflect the wider demographics of a species/population. This leads to a fundamental division between the remaining tools for investigating migration rate: those for investigating the degree of population admixture (typically for evolutionary, or population genetic related questions) and those used for investigating temporal, or recent migration (of more relevance to conservation/ecological questions), although, this is not a firm division.

One of the more widespread and evolutionary styled approaches for investigating population admixture is the coalescent. This is employed in: migrate/lamarc (Beerli & Felsenstein, 1999; 2001; Kuhner, 2006; Beerli & Palczewski, 2010), genetree (Bahlo & Griffiths, 2000), mdiv (Hey & Nielsen, 2004; Nielsen & Wakeley, 2001), ima/ima2 (Hey, 2010; Hey & Nielsen, 2007), diyabc2 (Cornuet et al., 2014), abctoolbox (Wegmann et al., 2010), and beast /beast2: (Bouckaert et al., 2014; Ewing et al., 2004; Ewing & Rodrigo, 2006). The coalescent can be used to simulate genealogies to create the probability distributions of the parameters of interest (Kuhner & Smith, 2006), or as a model description of the sampled populations (providing the population parameters for estimation; Nielsen & Wakeley, 2001). The coalescent is especially helpful for describing complex population structures, for example, as described in human populations (Bhatia et al., 2011). Humans have a hierarchical population structure that leads to increased allelic correlations among closely related subpopulations. This introduces biases into the migration estimates. Hence, incorporating hierarchical population structure is necessary to provide accurate estimates, as is available in ima (Hey, 2010; Hey & Nielsen, 2007; Nielsen & Wakeley, 2001; Sethuraman & Hey, 2016) and mimar (Becquet & Przeworski, 2007). These tools use phylogenetic structures to inform on population structure; ima estimates the genealogical structure of the population from sequence data from multiple loci (Sethuraman & Hey, 2016), while MIMAR uses a phylogenetic structure to identify the ancestral loci states (it required the presence of an outgroup—unfortunately MIMAR is no longer maintained; Becquent & Przeworski, 2007).

Alternatives to the above involve methods using allelic, or genotype frequencies with alternative inferences: for example, the use of method of moments estimators with genotype frequencies in estim (Vitalis & Couvet, 2001) for calculating likely migration rates, the use of Mantel tests in adgenet to test for isolation between subpopulations (Jombart, 2008), or the traditional calculation of F_ST_ statistics available in a wide range of tools described here (i.e., arlequin, pegas, popgene—Excoffier & Lischer, 2010; Paradis, 2010; Yeh & Boyle, 1997). A final consideration for users is the ease with which various tools can be employed to analyze HTS data, of those programs previously mentioned diyabc and migest/popcluster seem best suited for use with computing clusters (Cornuet et al., 2014; Wang, 2014). Alternatives such as pegas and adegenet may also read in HTS data, but are limited in terms of the number of loci to an order of around 10^5^, which may not be suitable for all HTS studies.

### 3.3 Effective population size and drift

Drift describes fluctuations in allelic frequencies caused by random processes in populations with finite size and it is rather difficult to estimate directly. Instead, the magnitude of drift is described by the effective population size (N_E_), both being negatively correlated with each other (Wang et al., 2016). N_E_ represents a key parameter in conservation and evolutionary biology (Charlesworth, 2009; Habel et al., 2014; Husemann et al., 2016; Lanfear et al., 2014; Luikart et al., 2010) and a variety of methods have been developed to estimate different types of N_E_ in natural populations (Barker, 2011; Luikart et al., 2010; Palstra & Ruzzante, 2008; Wang et al., 2016). In conservation biology, N_E_ is used as a measure of the susceptibility of a population to stochastic processes and inbreeding and hence can estimate the viability of a population (Hare et al., 2011). Besides the different types of N_E_, one can also distinguish between methods estimating the contemporary population size and those estimating demographic changes over time.

2mod was the only software found to directly estimate the presence of drift, which, however, is no longer supported and the interpretation of results was not intuitive (Ciofi et al., 1999). However, for many conservation applications and management decisions the contemporary N_E_ is a more important parameter. Its estimation usually involves a single panmictic population; deviations from the “optimal” population, for example, resulting from migration (e.g., Beerli & Palczewski, 2010), overlapping generations (Coombs et al., 2012; Waples et al., 2014), population subdivision (Ryman et al., 2014) and lack of clearly defined units in continuously distributed populations (Neel et al., 2013) lead to problems for estimating N_E_. Some of these limitations have been addressed conceptually or practically (Nunney, 2016; Wang et al., 2016; Waples et al., 2014), and some solutions have been implemented in software packages (Wang & Whitlock, 2003).

Estimates for the effective population size (N_E_) are broadly grouped into three categories (Wang et al., 2016): the variance N_E_, which is estimated from fluctuations in allele frequencies in genetic times series; the inbreeding N_E_, representing an estimate of N_E_ derived from linkage between sampled loci; and the coalescent N_E_, that either uses simulations, or estimates for branch lengths to infer N_E_. Variance N_E_, and some of the coalescent N_E_ methods, require sampling of the same population at least twice at different points in time, preferably several generations apart. This represents a main limitation, as such samples are rarely available in nonmodel taxa and organisms with long generation times (Habel et al., 2014).

Estimates of variance N_E_ can, for example, be obtained using tempofs (Jorde & Ryman, 2007), a moment‐based estimator with relatively low accuracy (Wang et al., 2016). Similarly, mlne and gone represent moment‐based inference methods for variance N_E_ (Wang, 2001; Wang & Whitlock, 2003), the latter providing a modification to adjust for age structure in populations (Coombs et al., 2012). However, as gone requires estimates of age‐specific survival and birth rates, it is difficult to apply to many datasets (Coombs et al., 2012). mlne in addition has the capability to estimate migration rates. Several other tools, such as tm3—(Berthier et al., 2002), tmvp—(Beaumont, 2003), and cone—(Anderson, 2005), also use temporal sampling, but estimate coalescent N_E_. Overall, for most programs estimating variance N_E_, the main limitation is the restriction in estimation accuracy to populations with small effective sizes (and the computationally intensive nature of the approaches). The r‐package nd (Hui & Burt, 2015) overcomes some of these limitations (to the applicable population sizes) by using a hidden Markov model to reduce computational load and raise the upper bounds (of N_E_) to several million individuals. mcleeps (Anderson et al., 2000) implicitly involves a Markov Chain and uses a Monte Carlo algorithm to overcome the computationally intensive nature of generating probability distributions for the effective population size. Despite the complexities involved, the temporal method makes fewer assumptions and is considered more robust for real populations (Wang et al., 2016).

Inbreeding N_E_, based on the linkage disequilibrium (LD) between sampled loci, is most commonly estimated using ldne (Waples, 2006; Waples & Do, 2008) and only requires a single temporal sample. Recently, extensions of the LD method have been developed to allow the estimation from many loci across the genome (Waples et al., 2014) and to incorporate the effects of linkage for genomic data, which is implemented in linkne (Hollenbeck et al., 2016). Further, the LD method has been implemented in snep (Barbato et al., 2015) to estimate recent N_E_ trajectories from genome‐wide SNP data.

Alternative single sample estimators are the heterozygosity excess method, which employs the relationship between the N_E_ of a parental population and the amount of heterozygosity excess in the offspring population (Luikart & Cornuet, 1999; Pudovkin et al., 1996; Robertson, 1975; Wang, 1996; Wang et al., 2016) as implemented in n_e_estimator (Do et al., 2014), or the sibship method, which uses inferred sibship/parentage frequencies as implemented in colony (Jones & Wang, 2009). The heterozygosity excess method, however, does not seem to perform well on empirical data (Wang et al., 2016).

The coalescence N_E_ is based on the assumption that two random gene copies will coalesce with a chance 1/2 N within one generation (Wakely & Sargsyan, 2009; see Wang et al., 2016 for a summary). The theory has been implemented in several software packages, such as tm3—(Berthier et al., 2002), tmvp—(Beaumont, 2003), and cone—(Anderson, 2005) which all use temporal sampling. These programs use maximum likelihood or pseudo‐likelihood to estimate coalescent N_E_ (for a more in depth review, see Wang et al., 2016). As many of the other methods, these tools are performing better with small N_E_ when drift is strong and have problems estimating N_E_ in large populations. Further, they are computationally relatively demanding and are limited to smaller datasets.

As with the other parameters, Bayesian approaches have brought more flexibility and allow more complexity for parameter estimation. While it is often not clear which type of N_E_ (inbreeding, variance, or coalescent) is estimated, the Bayesian approaches often not only estimate N_E_, but often trace changes in N_E_ over time. ABC methods (Csilléry et al., 2010) allow the implementation of demographic models and are now implemented in a variety of programs (WFABC—Foll et al., 2015; PopABC—Lopes et al., 2009; DIYABC—Cornuet et al., 2008; ABCtoolbox—Wegmann et al., 2010; and the abc R‐Package—Csillery et al., 2012), several of which employ the coalescent (Cornuet et al., 2008; Lopes et al., 2009). Yet, so far, ABC models have not been evaluated in comparison to more classic N_E_ estimation methods (Wang et al., 2016), although comparisons among various ABC implementations have been made. The issue of limited numbers of implementable summary statistics in traditional ABC has been solved using a kernel‐based approach (Nakagome et al., 2013). Kernelization allows for an increased number of summary statistics, while maintaining high performance. Other alternatives include implementing machine learning methods to avoid problems with the high number of dimensions in posterior estimation (Cornuet et al., 2014). Additionally, Markovian models are gaining attention in inferring historic population sizes from whole genome sequences. The initial pairwise sequential Markov chain model (PSMC, Li & Durbin, 2011) has been improved by the multiple sequential Markov chain model (MSMC, Schiffels & Durbin, 2014); a further alternative is provided by the dical model, which represents a generalization of the PSMC model applicable to multiple sequences (Sheehan et al., 2013). A more empirical distinction can be made with WFABC, as it is the only program that does not assume neutrality and enables the estimation of both N_E_ and S, potentially even allowing estimation of N_E_ when S = 1 (Bollback et al., 2008; Foll et al., 2015; Malaspinas et al., 2012; Mathieson & McVean, 2013).

Several studies have aimed to evaluate the performance of different methods to estimate N_E_ from genetic data. However, no gold standard has been reached, as performance strongly depends on the demographics of the studied populations. Many studies find that temporal estimates (variance N_E_) tend to be larger than single sample estimates (inbreeding N_E_) (Barker, 2011; Husemann et al., 2015; b). Different single sample estimators, in turn, frequently yield relatively congruent results (Àlvarez et al., 2015), but can prove sensitive to the study system (Holleley et al., 2014; Gilbert, Whitlock & Lotterhos, 2015). Hence, it remains important to use different estimators within the same study to gain confidence in the estimates. This is facilitated by the implementation of multiple estimators in single commonly used programs such as neestimator, which can accommodate more than 45,000 di‐allelic SNPs in the current version and hence is applicable to a wide variety of datasets.

### 3.4 Selection

In tests for selection, two different distinctions can be made in the applicability and approaches used. The first distinction that we make here is in the applicable use of the methods: those traditional statistics that are appropriate for single loci, and those for HTS datasets. The second distinction we make is within the analyses for HTS datasets: We distinguish between those that involve generating estimates of F_ST_ across populations and the genome, compared to those analyzing sequences for selective sweeps (looking for extended regions of homozygosity in the genome sequences).

In traditional population genetic analyses, Watterson's theta provides a measure of genetic diversity (Watterson, 1975), that can allow for a limited approach to testing selection through comparisons of genetic diversity with the pairwise estimate (Pi). These two measures are used within Tajima's D for estimating selection; this test essentially compares the distributions of variants quantifying either an excess, or dearth of rare variants (Tajima, 1989). Hence, deviations in Tajima's D can be used to infer different types of selection. However, this statistic is strongly influenced by demographic factors, such as population expansions, bottlenecks, and contractions. Two similarly traditional tests offer solutions to these problems, both dependent on the availability of additional information. The HKA test (Hudson, Kreitman & Aguade, 1987) uses information from multiple loci and species (a minimum of two), assuming that under neutral theory divergence between the loci should be equivalent to the difference in polymorphism. The McDonald–Kreitman (MK) test predominantly does not use multiple loci (although variations exist), but does require the use of an outgroup as it uses comparisons of sequence divergence and diversity to estimate deviations from neutrality and directionality (Eyre‐Walker, 2006). These tests are widely available within several packages (mega6—Tamura et al., 2013; popgene—Yeh & Boyle, 1997; Tajima's D in arlequin—Excoffier & Schneider, 2005; and its namesake in mk—Egea et al., 2008). Differentiation between these implementations are minor, the larger differences lie within the data formats. For mega, file conversions are necessary and are available within sequence viewing/analysis tools (as well as within mega for a large variety of formats). arlequin and mk can ultimately read in sequence formats (mk from multiple loci). In contrast popgene primarily reads in a variety of different allele/marker encodings (as does arlequin), providing for analysis of dominant and codominant markers.

Differences between these traditional methods of estimating selection involve the range of genetic sequence information available. Typically, they require the use of sequence data and are not appropriate for use with dominant markers. Tajima's D requires data from a single population, while mk requires the presence of a single suitable outgroup (preferably a sister species). The HKA test is arguably more demanding in the requirement of needing sequences from at least two loci. All of these tests run into problems with either longer sequences or HTS datasets, in that they are susceptible to producing elevated levels of error (Andolfatto, 2008). To reduce the rates of error from a lack of control of the influence of recombination on the test statistics, sliding window approaches can be employed (Nielsen et al., 2005); although difficulties in interpreting significance may remain.

To deal with either longer sequences or HTS datasets, several approaches exist. One of the popular “families” of approaches is derived from the Lewontin–Krakauer (LK) test (Lewontin & Krakauer, 1973). This uses the F_ST_ statistic calculated across multiple loci and populations to test for deviations in the estimates. These deviations are then used to infer selection through outlier analyses (Bonhomme et al., 2010). While these methods are appropriate for testing recent and ongoing selection (as well as variation in selection between populations), they are also generally beset by problems arising from correlations in F_ST_ due to hierarchical population structure (typical for human populations) and linkage between loci. The tools currently available employ a variety of different techniques to circumvent or avoid these issues. FLK (Bonhomme et al., 2010) uses a hierarchical estimate of the population structure through a kinship matrix to avoid interference from the population structure. To estimate this, flk requires the presence of a population that can act as an outgroup (it can also incorporate previous estimates of genetic distance). hapflk also avoids interference from population structure and is more robust against interference on the estimates from linkage, yet requires a known pedigree for assessing population structure (Fariello et al., 2013). In comparison, treeselect has no requirement on data informing on the population structure; unfortunately, the marker sets for analysis must be shared across all populations (Bhatia et al., 2011). A solution that might be limiting for species, where strong population differentiation might exist. The other methods in this LK family mainly differentiate through the techniques of inference. detsel (Vitalis et al., 2003) uses simulated distributions of differentiation using allele counts across multiple populations to produce a null distribution (and avoid issues with independence between samples), while hacdivsel (Carvajal‐Rodriguez, 2017) uses two complimentary approaches for inferring selection (haplotype and outlier based). hacdivsel is also one of the few tools that accepts sequence data for analysis (most of the alternative approaches require some processing of HTS data before analyses can be run). In comparison, the LK‐based outflank (Whitlock & Lotterhos, 2015) uses a matrix of F_ST_ values as its input data and analyses of selection are run on these values.

F_ST_ approaches are also used by other programs, of those previously mentioned arlequin is one of the more commonly cited tools where an F_ST_ test is implemented (Excoffier & Lischer, 2010). arlequin, along with bayescan, bayesfst, bayenv, and selestim all use outlier based tests for inferring the presence of selection with F_ST_ (respectively, Balding, 2003; Foll & Gaggioti, 2008; Gunther & Coop, 2013; Vitalis et al., 2014). Alternatively, eigenvector based regression models can be used in the tools eigengwas or pcadapt (Chen et al., 2015; Luu et al., 2017). For the outlier tests, all the tools use allelic count data from multiple populations for generating F_ST_ estimates. Differentiating itself from the other tools bayenv can optionally include the degree of covariance between the environments of the different populations to inform predicted correlations in allelic frequencies (Gunther & Coop, 2013). Otherwise, differentiation between these tools is largely limited to the possible input data: The majority can use SNP counts (biallelic in bayenv, arlequin, bayesfst, selestim; and multiallelic with bayescan), bayescan can use dominant markers, while selestim can use read counts in place of allele counts for input (Foll & Gaggioti, 2008; Vitalis et al., 2014).

For more information regarding differences between these available tools, a variety of reviews are available. Most of these comparisons have been performed upon the publication of a new method: Vitalis et al. (2014), for example, demonstrated the advantages provided by selestim against bayescan. DeGiorgio et al. (2014) in a more stark contrast investigated the ability of tools to estimate balancing selection, demonstrating that ballet (designed purely for testing balancing selection) provided improvements beyond those present in hapflk compared to selestim (Fariello et al., 2013).

The second “family” of approaches we consider are the selective sweep‐based approaches. These can be further subdivided into the methods that can detect hard and soft sweeps. Hard sweeps define those situations where a new allele has reached fixation in a population, leading to extensive regions of homozygosity. The common framework for this test is haplotype homozygosity statistics (HH). This statistic identifies the probability that a core haplotype is identical by descent between two randomly chosen chromosomes (Sabeti et al., 2002). The second approach is suitable for detecting selection on preexisting mutations or incomplete selective sweeps (soft sweeps), through analyzing distortions in the allele frequencies, away from those expected under neutrality. These analyses (particularly the hard sweep tests) are generally predicated on population‐wide selection. Historical and recent selection events can be tested by either of these approaches, whereas ongoing processes are ascertainable only through tools developed for detecting soft selective sweeps.

Among those programs analyzing HTS data for hard sweeps, sweep was one of the earlier ones employing the extended haplotype homozygosity (EHH) statistic and using a coalescent model to generate parameter probabilities (Sabeti et al., 2002). All of the later models that use the HH statistics (selscan, rehh, ihs, xpehh, and hapbin) incorporate the long range haplotype (LRH), integrated HH (iHS), and cross‐population (XP‐EHH) statistics (respectively, Szpiech & Hernandez, 2014; Gautier & Vitalis, 2012; Voight et al., 2006; Pickrell et al., 2009; and Maclean et al., 2015). Differences between these programs involve changes to the algorithms in the utilization of modern technologies to improve performance, reducing run times (Szpiech & Hernandez, 2014). selscan, for example, offers significant speed improvements over rehh, ihs, and xpehh through multi‐threading (Szpiech & Hernandez, 2014), while hapbin reportedly demonstrates even greater improvements over selscan (~3,000 fold speed increase, Maclean et al., 2015). It should be noted that both hapbin and selscan require additional mapping information and processing for the loci, when compared to the R‐package rehh.

From the software detecting hard sweeps, only selscan also offers methods to analyze soft sweeps, using another recently developed statistic (nS_L_, Szpiech & Hernandez, 2014). This statistic being originally developed for the program ns_l_ by Ferrer‐Admetlla et al. (2014), provides more reliable estimates when variation in recombination rates is present in the data and fixation of alleles in haplotypes is not expected (ns_l_ itself is no longer supported). Other techniques for analyzing soft sweeps include investigations of the distortion of linkage disequilibrium (developed from the statistics of Kim & Nielsen, 2004), as provided in omegaplus (Alachiotis et al., 2012), or using site frequency spectra (the distribution in allele frequencies at a set of loci), as available in the sweepfinder programs (including sweed: Nielsen et al., 2005; DeGiorgio et al., 2016; and Pavlidis et al., 2013). While the files describing the input data should all follow the same format (frequencies of a binary description of alleles/marker data), sweed offers distinct speed advantages in regards to HTS data (Pavlidis et al., 2013). This approach carries the cost of requiring increased preprocessing of data from the raw reads. However, it can offer advantages compared to earlier approaches for detecting hard sweeps. Excluding selscan, data input is similar for all of these programs: they require allele frequency data. The sweepfinder programs can also utilize data on background selection to improve estimates.

### 3.5 Citation bias

In addition to the short reflection and documentation of software developments to estimate population genetic (micro‐evolutionary) parameters, we were interested in the usage bias of such programs. As no direct estimate of user numbers is available for many of the software packages, we used the frequency with which the programs are cited as a proxy of user frequency. With the citation records available on ISI databases, we could gather a fairly standardized measure for the frequency of program/software usage. Of the 101 programs and scripts investigated (Table 1), some programs were not published in peer‐reviewed journals. Furthermore, of those published, only those before 2014 were investigated. The pattern of growth in the citation record was visually inspected for this subset (plus the total number of citations for the four most cited programs: mega, arlequin, dnasp, and structure, Figure S1). As a consequence the geometric mean was used for measuring the average number of citations per year (for all relevant tests).

**FIGURE 1:**
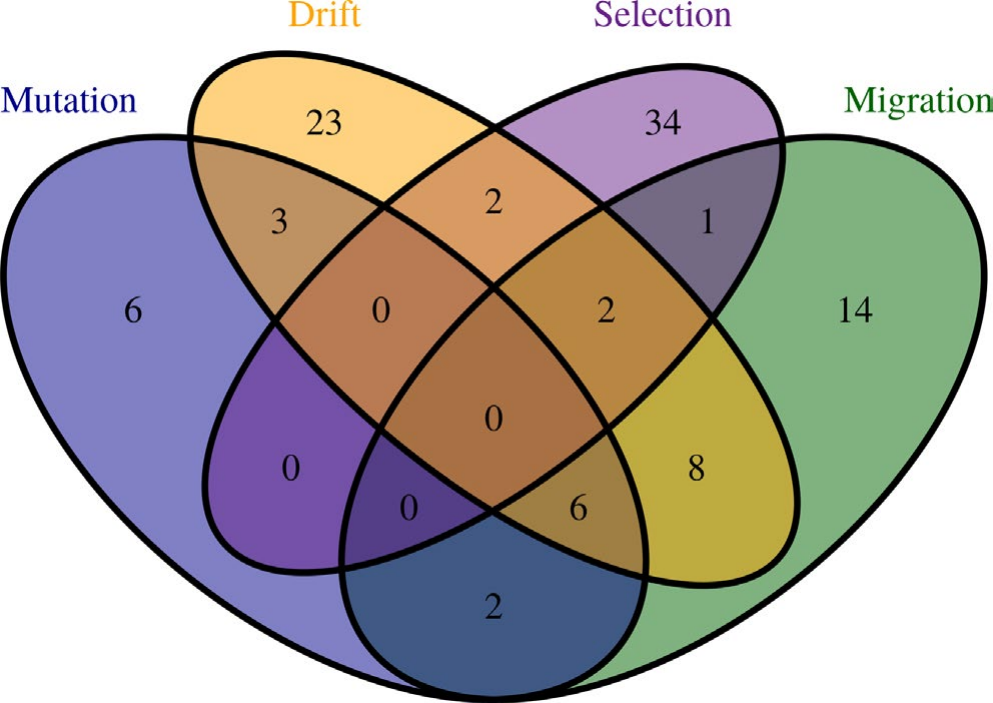
Distribution of the programs estimating the various population genetic parameters. While the software suites for estimating mutation, gene flow (migration) and drift can be observed to follow the same distributions (hypergeometric tests, probability of coming from the same distribution *p* > .5), the same cannot be said of selection (hypergeometric test, *p* < 10–08). This may suggest that the development of software for detecting selection comes from a different research community compared to the other parameters

**FIGURE 2:**
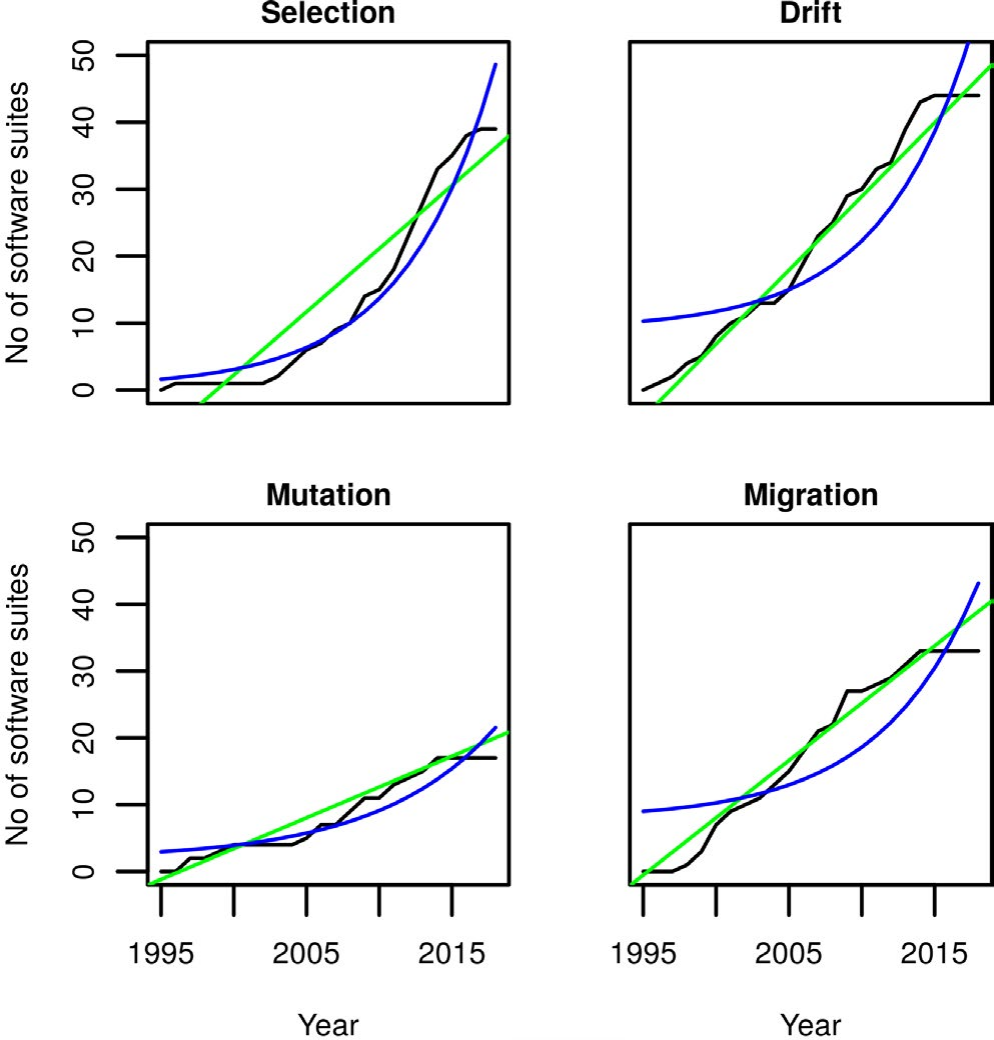
Comparison of linear (green) to exponential (blue) growth of the number of software suites for estimating the various parameters, plotted against the data (black). The data for selection, fit to the nonlinear model better than to the linear (linear‐MSS = 14.42, nonlinear‐MSS = 7.88; *n* = 38), although this difference is considerably weaker than for the other parameters. For mutation (linear‐MSS = 2.39, nonlinear‐MSS = 6.74; *n* = 17), migration (linear‐MSS = 3.37, nonlinear‐MSS = 31.43; *n* = 33) and drift (linear‐MSS = 2.48, nonlinear‐MSS = 26.14; *n* = 44), the linear models all fit considerably closer to the observed data. These differences are taken to illustrate a more rapid rate of growth in the development of software analyzing/estimating selection, over drift, mutation, and migration

We found clear differences between the programs developed to analyze the different parameters: Generally, more programs can be found to estimate selection and migration. Furthermore, a more distinct research community appears to exist for investigating selection, than for the other parameters (there is an absence of overlap of programs estimating selection with the other micro‐evolutionary parameters (hyper‐geometric tests, *p* < 10^−08^ for selection, compared to *p* > .5 for migration, mutation, drift; Figure 1)). This may also translate through to the observed different ages/growth in the number of programs for testing the different parameters (Figure 2), with programs measuring selection undergoing exponential growth (the numbers for the other parameters showing a linear increase over time). This points toward a growing interest in estimating selection in the last years.

In an attempt to disambiguate the potential role of measured factors on the citation bias, Kruskal–Wallis rank sum tests were performed over the platforms and parameters estimated. While no significant effects were observed for the computing platforms (*x* ^2^ = 8.86, *df* = 7, *p* > .2, *N* = 86), differences were observed according to the number of estimated parameters (*x* ^2^ = 23.96, *df* = 10, *p* < .01, *N* = 86). These differences, however, were due to either single programs, or suites from a single group/development (respectively, arlequin v.3 and v.3.5, and beast v.1, v.1.7 and v.2). Dropping these records as outliers, no significant differences were observed (*x* ^2^ = 15.19, *df* = 8, *p* > .05, *N* = 81). For all parameters, though, there was a high disparity in the distribution of citations: over all programs more than half the citations (>62%) belonged to the four most cited (top five percent of the most cited) programs; respectively, these are structure, mega6, arlequin v.3, and dnasp (all of which have been in development beyond the average development time observed here, 2008).

**FIGURE 3:**
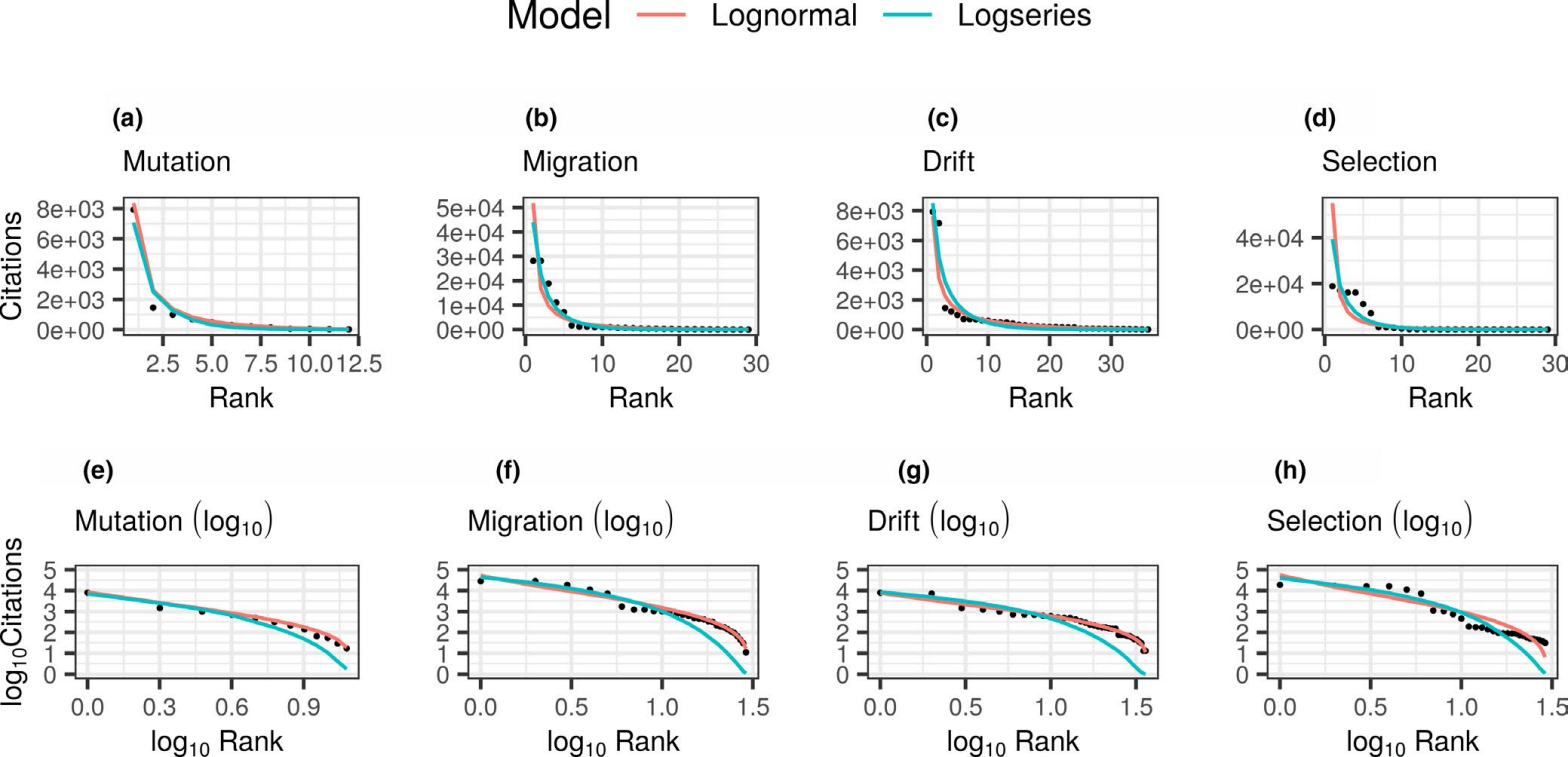
Log‐normal and log‐series plots of the citation records for the different micro‐evolutionary parameters. In these series, the number of citations reported on ISI web of knowledge site are displayed as dependent on the citation rank. These results are reported as raw counts (a–d), or as plots of the log (to base 10, e–h) for both the dependent and independent variables. From all of these plots, the log‐normal model fits better to the observed data, independent of the parameter the programs estimate. Generally, the log‐series fits data with inflated single observations, here single citations, a situation that is unlikely to be common here. A situation that does not reflect the data where the greatest deviation from the log‐normal was observed (d, h)

**FIGURE 4:**
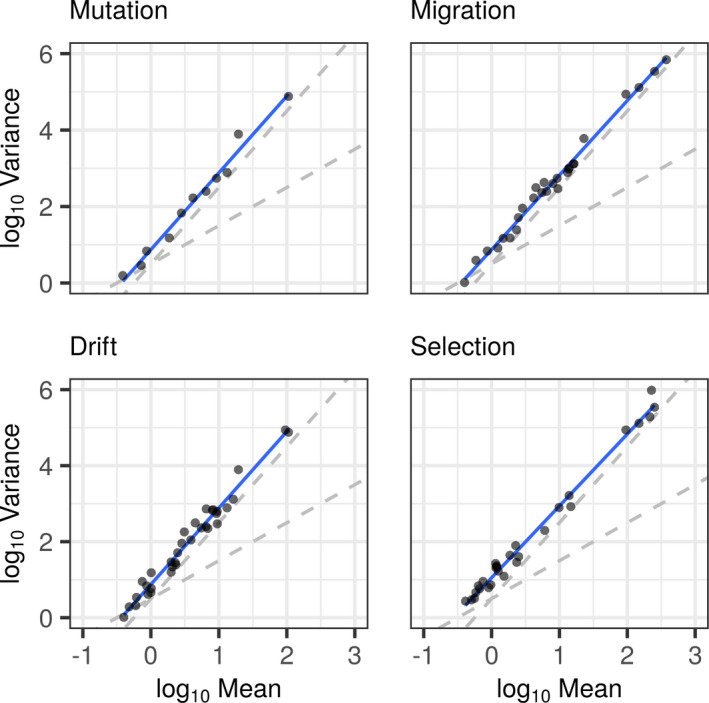
Taylors power law graphs illustrating the relationship between the variance and mean in distribution of citations. Here, the pattern of the citation bias is plotted using the log of the means (to base 10) as a predictor of the variance. With the slope of the temporal variance and mean equal to 2 (the steeper dashed line, in all four graphs), the process of bias in citations follows a simple power law where the variation in citation follows previous frequencies of citation. Hence, alternative factors affecting the distribution of citation bias can be discarded. This relationship is observed for all four population genetic parameters (Mutation, upper‐left; Migration, upper‐right; Drift lower‐left; and Selection, lower‐right)

With such a highly skewed distribution, we fitted log‐normal and log‐series models to our citation dataset (Figure 3). Irrespective of the parameter that the software or program is designed to estimate, the citation records strongly fitted these log‐models. These distributions appear to fit the ecological law described by Taylor (Taylor, 1961) in Figure 4, whereby the frequency with which a program is observed is based on random processes, namely, its previous observed frequency. We tested this using a log‐regression model (*b* = 1.93 ± 0.03 (*SE*), *df* = 84, *N* = 86, *p* < 2 × 10^−16^; log(var)~log(mean)), which shows a value deviating slightly from 2.0. This indicates that while there may be some processes other than Taylor's power law influencing the usage of the software tools, a simple exponential model of growth provides a powerful explanation of our observed results. Where the number of citations is the best predictor for future citations. A fact that is not due to an over abundance of very infrequently cited tools as evidenced in Figure 3 (where better fits to log‐normal models are observed).

**FIGURE 5:**
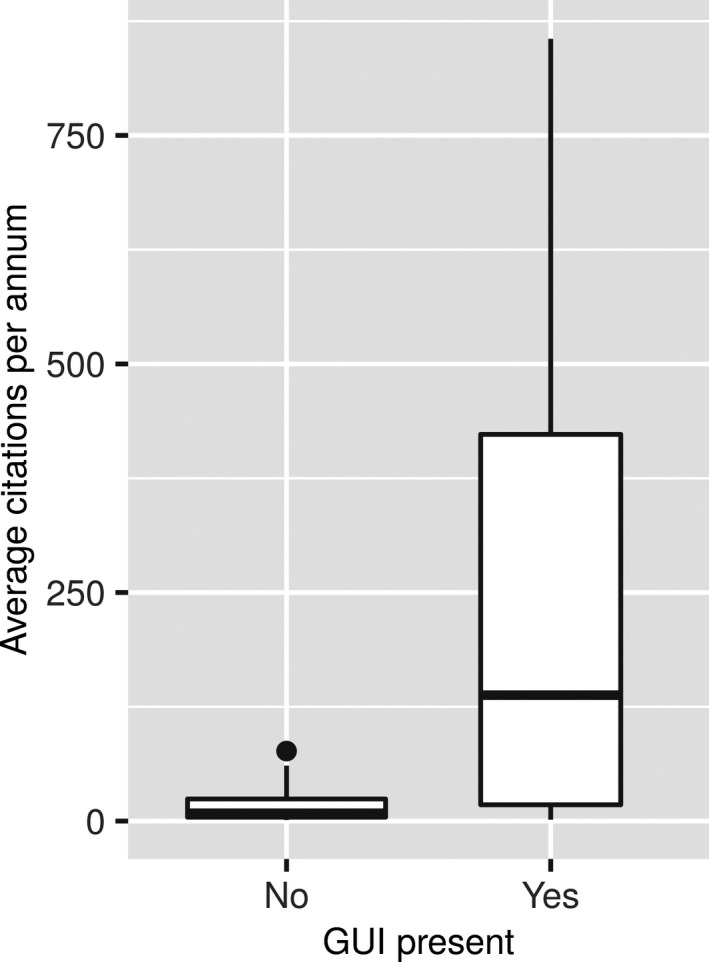
Effect of existence of a GUI on the citation rate. The differences in the geometric mean in citation rate according to the presence or absence of a GUI for software for estimating any of the four parameters considered here (mutation, drift, migration, or selection). Much greater range sizes are observed in the citation records for those software suites where a GUI has been developed, with this difference being highly significant (Wilcoxon rank sum test, *W* = 186, *p*‐value 4.8 × 10^−04^, *N* = 65)

To investigate whether this deviance from Taylor's power law is being influenced by other parameters, we analyzed the journal IF (where the program was published in) and presence/absence of a GUI. There was no effect based on the regression of the geometric mean of citations (to remove the impact of the life span of the program) on IF (Figure S2), using a robust linear model (R‐Cran MASS package: value = 0.74, *SE* = 0.42, *t*‐value = 1.76). Although it should be noted that this result is difficult to interpret, correlations between manuscript/software quality and journal IF can be expected. Whether the lack of observed significance for the IF indicates the lack of an effect for either role remains unclear (Figure S2). In contrast, a strong effect from the presence/absence of a GUI (Figure 5, Wilcoxon rank sum test, *W* = 186, *p*‐value <4.8 × 10^−04^, *N* = 65) suggests an important role for the “ease of use” for the adoption of programs within the biological community. Complications to this conclusion arise from the fact that some of the older suites have incorporated GUIs since their initial publication, as well as the development of some tools within previously existing frameworks (R‐Cran; R Core Team, 2018).

Investigating further factors that could underlie the distribution of software usage contains additional difficulties, including confounding factors for many hypotheses. This can be illustrated through the concept of “service provision” in the software “marketplace”. The “marketplace” represents the diversity of software and algorithms that are available to researchers, each individual program offering a service within the “marketplace”. With the advent of new technologies (as occurred with HTS), a range of new niches arose allowing for new/more “service provision” from new and existing software. Any early developments in such an area gain an advantage through a higher degree of “service provision”. This encourages further developments and software within the field, each subsequent development (excluding the expansion of niches), in turn, offering fewer novel services. Hence, the “service provision” of software depends on the population of preexisting software and cannot be considered temporally independent. Another factor difficult to measure is the degree to which researchers continue to utilize tools due to familiarity, as this repetition of use could also reflect the quality of the software. Similarly, the ability of software developers to gain attention from the research community may be related to the required investment from the users to develop the skills and data to employ the new software. This might also depend on the degree of “overlap” between the diversity of software tools that are available, for example, the wide range of programs providing LK tests for detecting selection, or F_ST_ variance tests for population size/drift. In cases of high overlap, the usability will play a major role compared to niches, which are only occupied by single, or few programs.

Stochastic processes have been shown to be present in other human systems, such as the use/success of Linux distributions (Keil et al., 2018) and law and order (Hanley, Lewis & Ribeiro 2016). While the frequency that a software tool is observed might still be related to the age/development of the tool, such data were not available in the current study. The presence of multiple articles and iterations of the different software tools additionally makes such analyses considerably more difficult. Despite this, one can ascertain distinctions between the different communities involved in software development (Figure 1) and factors leading to differences in citation frequency (Figure 5). Further investigations within the use of population genetics software within the scientific community may best be advanced through the collection of more data on the tools, or more thorough investigations of some of the specific communities. For instance, more recent studies on selection have employed multiple different statistics to identify candidate sites using Tajima's D, LK tests (hapflk) and the iHS (ihs) (Harpur et al., 2019). Investigation of the potential use of complementary approaches in population genetic analyses might be of much wider interest to the research community. This could be investigated through the robustness of tools, such as hacdivsel (Carvajal‐Rodriguez, 2017), that incorporate multiple statistics compared to tools that employ a single family of techniques.

## 4 CONCLUSIONS

A large assortment of software exists for the estimation of population genetic parameters; between these parameters the development environments appear to be distinct (Figures 1 and 2). (Although this should be taken with some caution as this may be the result of low usage frequencies observed before 2002/03.) While not every software suite offers unique functionality, the majority offer unique capabilities. A clear distinction for this can be found in the discussed software: differences in capability can be seen in genalex that estimates migration rates, and structure that analyses admixture and population stratification, assigning samples to populations. In contrast, arlequin offers the functionality of offering multiple different capabilities, that, however, are not unique and do not offer the user different capabilities. Despite the large number of available solutions, it appears, that in the majority of cases a much more limited range of software suites are actually utilized, potentially suggesting an under‐exploitation of a large variety of bioinformatic resources within molecular population genetics. Key to determining the degree of exploitation of the bioinformatic resources is the presence of a GUI, the only factor identified to predict high citation frequencies. The range of under‐exploitation is, however, observed for all the population genetic parameters investigated here (Figure 3). The effect potentially being more prevalent for those software suites estimating selection and migration, as greater ranges in citation frequencies were observed for these parameters.

Future studies should also implement the analyses of custom scripts, which are more frequently used in the advent of genomic studies. Further, hybridization and recombination have been established as important additional evolutionary forces and need to be included in future analyses of population genetic software.

## CONFLICT OF INTEREST

The authors declare that the research was conducted in the absence of any commercial or financial relationships that could be construed as a potential conflict of interest.

## AUTHOR CONTRIBUTIONS

**Jonathan Kidner:** Data curation (equal); formal analysis (equal); investigation (equal); methodology (equal); software (equal); visualization (lead); writing–original draft (equal); writing–review and editing (equal). **Panagiotis Theodorou:** Conceptualization (equal); data curation (equal); formal analysis (equal); investigation (equal); methodology (equal); software (equal); supervision (equal); visualization (supporting); writing–original draft (equal); writing–review and editing (equal). **Jan O. Engler:** Data curation (equal); formal analysis (supporting); investigation (equal); methodology (equal); software (equal); writing–original draft (equal); writing–review and editing (equal). **Martin Taubert:** Data curation (equal); methodology (equal); supervision (equal); writing–original draft (supporting); writing–review and editing (equal). **Martin Husemann:** Conceptualization (lead); data curation (equal); formal analysis (supporting); funding acquisition (lead); investigation (equal); methodology (equal); project administration (lead); software (equal); supervision (equal); writing–original draft (equal); writing–review and editing (equal).

## Supporting information

Fig S1

Fig S2

Appendix S1

## Data Availability

All datasets generated and analyzed for this study are provided within the manuscript and Appendix S1. All the data used are additionally available on Dryad with the accession number/address https://doi.org/10.5061/dryad.0p2ngf220
