# Supplementary figures and images for: A brief history and popularity of methods and tools used to estimate micro‐evolutionary forces
Source: Ecol Evol. 2021 Sep 16;11(20):13723–43. doi: 10.1002/ece3.8076 (PMC8525119; doi:10.1002/ece3.8076)

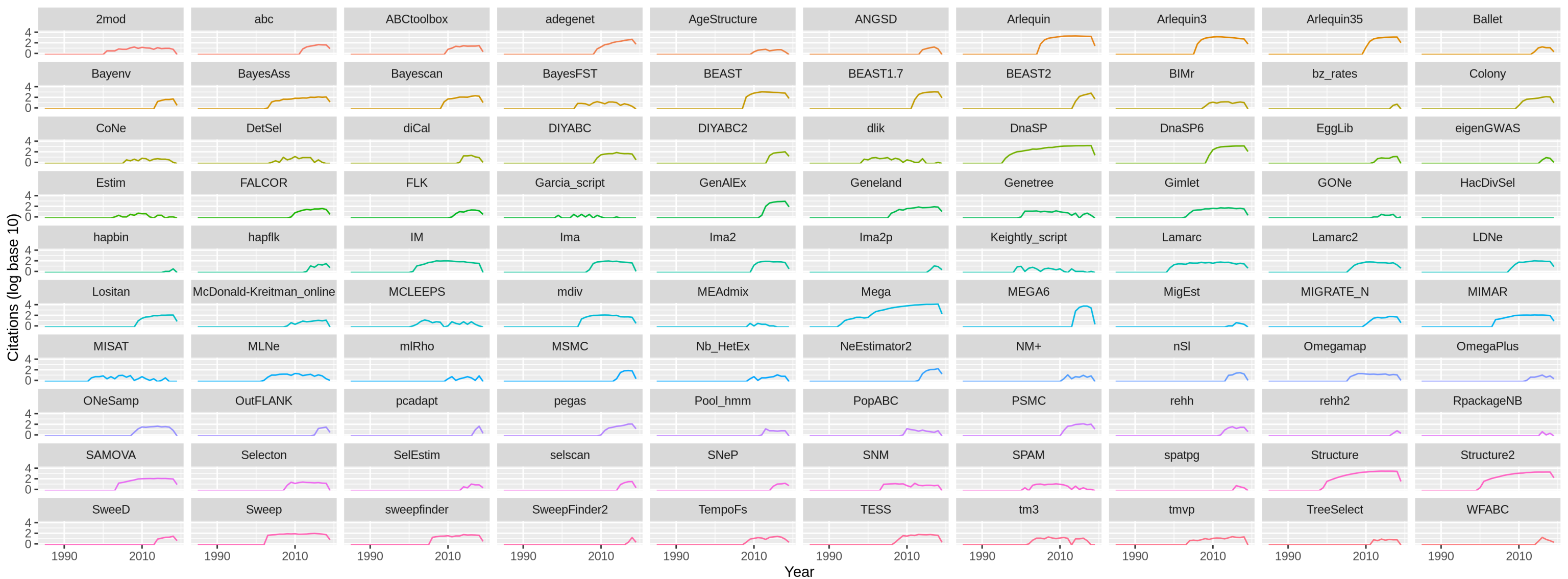

Supplement: Supplementary file 1 — Fig S1 [file ECE3-11-13723-s001.pdf]

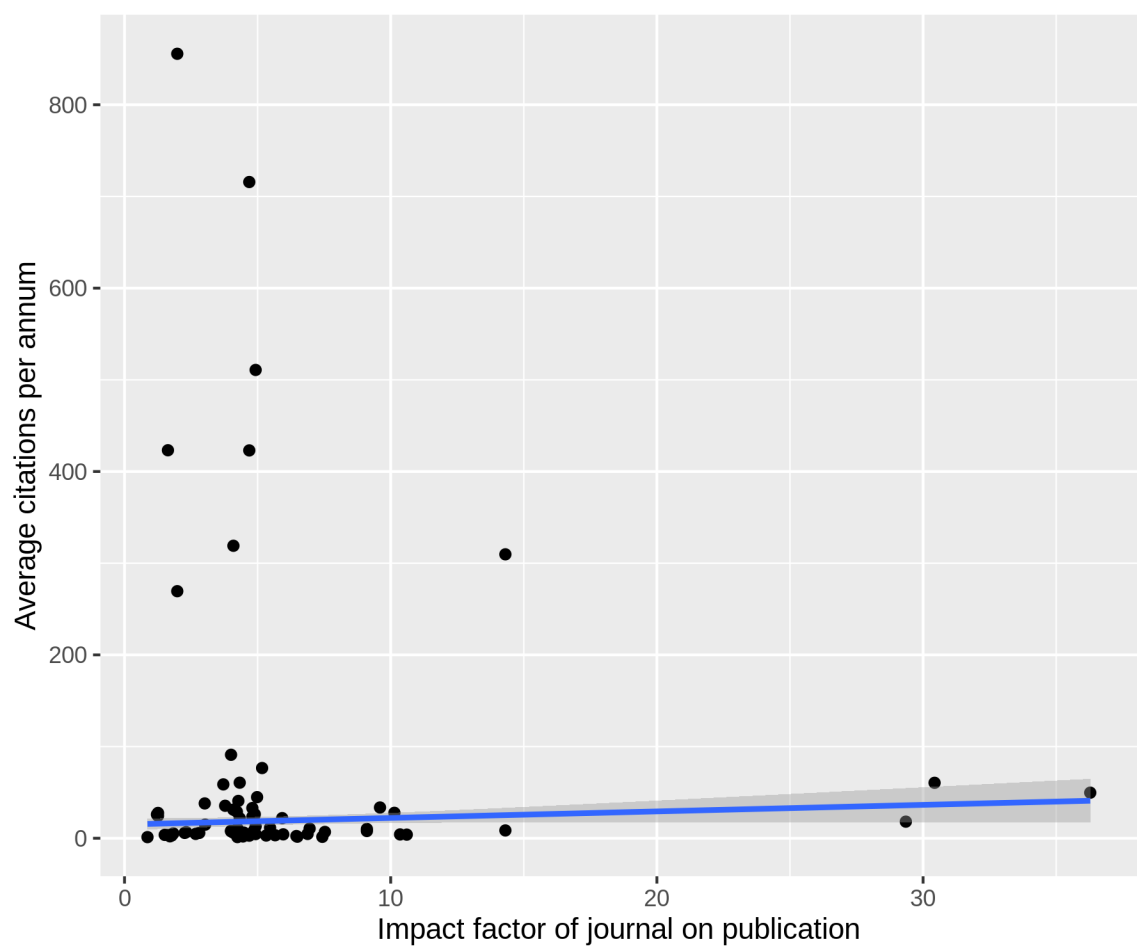

Supplement: Supplementary file 2 — Fig S2 [file ECE3-11-13723-s002.pdf]
