# Supplementary material for: A brief history and popularity of methods and tools used to estimate micro‐evolutionary forces
Source: Ecol Evol. 2021 Sep 16;11(20):13723–43. doi: 10.1002/ece3.8076 (PMC8525119; doi:10.1002/ece3.8076)
Supplement: Supplementary file 3 — Appendix S1 [file ECE3-11-13723-s003.pdf]

Table 1: The full table of software included in the analyses. The columns defined by μ, M, N_E and S respectively refer to the parameters: Mutation, Migration, Effective Population Size and Selection. IF indicates the impact factor at the time of publication, while GUI indicates the presence of a Graphical User Interface. Indication of a softwares inability to analyse a parameter is indicated by absences, as is its operation in computing environments

Program Name	Year	u	M	NE	S	Windows	Mac	Linux	IF	GUI	Reference	DOI	Link
2mod	1999			yes		yes			NA	NA	Ciofi et al., 1999	10.1098/rspb.1999.0918	http://www.maths.bris.ac.uk/ mamab/ software/
abc	2012		yes	yes		yes	yes	yes	5.924	No	Csillery et al., 2012	10.1111/j.2041-210X.2011.00179.x	https://cran.r-project.org/web/packages/abc/index.html
ABCtoolbox	2010		yes	yes			yes	yes	3.029	No	Wegmann et al., 2010	10.1186/1471-2105-11-116	https://github.com/sakeel/ABCtoolbox
adegenet	2008		yes			yes	yes	yes	4.328	No	Jombart 2008	10.1093/bioinformatics/btn129	http://adegenet.r-forge.r-project.org/
AgeStructure	2010			yes		yes			5.659	NA	Wang et al., 2010	10.1111/j.1558-5646.2010.00953.x	http://www.zsl.org/science/ software/agestructure
ANGSD	2013				yes			yes	2.672	No	Korneliussen et al., 2013	10.1186/1471-2105-14-289	github.com/ANGSD/angsd
Arlequin3	2005		yes		yes	yes			NA	Yes	Excoffier et al., 2005	NA	cmpg.unibe.ch/software/arlequin
Arlequin35	2010		yes	yes	yes	yes			1.631	Yes	Excoffier & Lischer, 2010	10.1111/j.1755-0998.2010.02847.x	http://cmpg.unibe.ch/software/arlequin35/
Ballet	2014			yes	yes			yes	7.528	No	DeGiorgio et al., 2014	10.1371/journal.pgen.1004561	www.personal.psu.edu/mxd60/software.html
Bayenv	2010				yes		yes	yes	4.866	No	Gunther & Coop, 2013	10.1534/genetics.113.152462	https://bitbucket.org/ tguenther/bayenv2
BayesAss	2003		yes			yes	yes	yes	4.276	No	Wilson & Rannala, 2003	NA	http://www.rannala.org/
Bayescan	2012				yes	yes	yes	yes	4.002	Yes	Foll & Gaggiotti, 2008	10.1534/genetics.108.092221	cmpg.unibe.ch/software/BayeScan
BayesFST	2004				yes	yes	yes	yes	2.261	No	Balding, 2003	10.1016/S0040-5809(03)00007-8	www.reading.ac.uk/Statistics/ genetics/software.html
BayPass	2015				yes	yes	yes	yes	4.644	No	Gautier 2015	10.1534/genetics.115.181453/-/DC1	www1.montpellier.inra.fr/ CBGP/software/baypass/
BEAST	2007	yes		yes		yes	yes	yes	4.091	Yes	Drummond & Rambaut, 2007	10.1186/1471-2148-7-214	http://beast.bio.ed.ac.uk/
BEAST17	2012	yes		yes		yes	yes	yes	10.353	Yes	Drummond et al., 2012	10.1093/molbev/mss075	http://beast.bio.ed.ac.uk/
BEAST2	2014	yes		yes		yes	yes	yes	NA	Yes	Bouckaert et al., 2014	10.1371/journal.pcbi.1003537	http://beast2.org/
BIMr	2008		yes			yes	yes	yes	4.002	Yes	Faubet & Gaggiotti, 2008	10.1534/genetics.107.082560	http://www-leca.ujf-grenoble.fr/moyens-techniques/logiciels/article/population-genomics-software?lang=frBIMr
bz_rates	2015	yes				yes	yes	yes	2.91	Yes	Gillet-Markowska et al., 2015	10.1534/g3.115.019836	http://www.lcqb.upmc.fr/bzrates
Colony	2009			yes		yes	yes	yes	1.259	NA	Jones & Wang, 2009	10.1111/j.1755-0998.2009.02787.x	http://www.zsl.org/science/software/colony
CoNe	2004			yes		yes			4.289	NA	Anderson, 2005	10.1534/genetics.104.038349	https://swfsc.noaa.gov/textblock.aspx?Division=FED&ParentMenuId=54&id=3436
DetSel	2013				yes	yes	yes	yes	1.707	No	Vitalis et al., 2003	10.1093/jhered/esg083	Github.com/cran/DetSel

– continued from previous page

Program Name	Year	u	M	NE	S	Windows	Mac	Linux	IF	GUI	Reference	DOI	Link
diCal	2013			yes		yes	yes	yes	4.866	NA	Sheehan et al., 2013	10.1534/genetics.112.149096	http://sourceforge.net/projects/dical/
DIYABC	2008		yes	yes		yes	yes	yes	4.328	NA	Cornuet et al., 2008	10.1093/bioinformatics/btn514	http://www1.montpellier.inra.fr/CBGP/diyabc/
DIYABC2	2014		yes	yes		yes	yes	yes	4.981	Yes	Cornuet et al., 2014	10.1093/bioinformatics/btt763	http://www1.montpellier.inra.fr/CBGP/diyabc/
dlik	1998			yes		yes			NA	NA	O'Ryan et al., 1998	10.1111/j.1469-1795.1998.tb00015.x	http://www.maths.bris.ac.uk/mamab/software/
DnaSP	2009				yes	yes			4.926	Yes	Librado and Rozas, 2009	10.1093/bioinformatics/btp187	www.ub.edu/dnasp/
EggLib	2014				yes	yes	yes	yes	2.808	No	De Mita & Siol, 2012	10.1186/1471-2156-13-27	egglib.sourceforge.net
EigenGWAS	2015				yes	yes	yes	yes	3.801	No	Chen et al., 2015	10.1038/hdy.2016.25	github.com/gc5k/GEAR/wiki
Estim	2001		yes	yes		yes			NA	NA	Vitalis & Couvet, 2001	10.1046/j.1471-8278.2001.00086.x	ftp://isem.isem.univ-montp2.fr/pub/pc/estim
FALCOR	2009	yes				yes	yes	yes	4.926	Yes	Hall et al., 2009	10.1093/bioinformatics/btp253	http://www.keshavsingh.org/protocols/FALCOR.html
FLK	2010				yes	yes	yes	yes	4.087	No	Bonhomme et al., 2010	10.1534/genetics.110.117275	https://qgsp.jouy.inra.fr/index.php? option=com_content&view=article&id=50&Itemid=55
Garcia_script	1998	yes				yes			0.863	No	García-Dorado & Marín, 1998	10.2307/2533860	available from the corresponding author
GenAlEx	2006		yes			yes	yes	yes	4.894	Yes	Peakall and Smouse 2006	10.1093/bioinformatics/bts460	http://biology.anu.edu.au/GenAlEx
Geneland	2005		yes			yes	yes	yes	1.219	No	Guillot et al., 2005	10.1111/j.1471-8286.2005.01031.x	https://i-pri.org/special/Biostatistics/Software/Geneland/index.html
Genetree	2000	yes	yes			yes		yes	1.833	NA	Bahlo & Griffiths, 2000	10.1006/tpbi.1999.1447	http://www.stats.ox.ac.uk/ griff/ software.html
Gimlet	2002			yes		yes			NA	Yes	Valiere, 2002	10.1046/j.1471-8286.2002.00228.x-i2	http://pbil.univ-lyon1.fr/software/Gimlet/gimlet%20frame1.html
GONe	2012			yes		yes			7.432	Yes	Coombs et al., 2012	10.1111/j.1755-0998.2011.03057.x	http://www.mybiosoftware.com/1-03-estimating-effective-size-ne-populations-overlapping-generations.html
HacDivSel	2017				yes		yes	yes	2.766	No	Carvajal-Rodriguez, 2017	10.1371/journal.pone.0175944	https://omictools.com/hacdivsel-tool
hapbin	2015				yes			yes	13.649	No	Maclean et al., 2015	10.1093/molbev/msv172	github.com/evotools/hapbin
hapflk	2013				yes		yes	yes	4.866	No	Fariello et al., 2013	10.1534/genetics.112.147231	https://pypi.org/project/hapflk/
IM	2001	yes	yes	yes		yes	yes	yes	4.803	NA	Nielsen & Wakeley, 2001	NA	https://bio.cst.temple.edu/ hey/software/software.htm
Ima	2007	yes	yes	yes		yes	yes	yes	9.598	No	Hey & Nielsen, 2007	10.1073/pnas.0611164104	https://bio.cst.temple.edu/ hey/software
IMa2	2010	yes	yes	yes		yes	yes	yes	5.51	No	Hey, 2010	10.1093/molbev/msp296	https://bio.cst.temple.edu/ hey/software
Ima2p	2015	yes	yes	yes		yes	yes	yes	5.298	No	Sethuraman & Hey, 2015	10.1111/1755-0998.12437	https://bio.cst.temple.edu/ hey/software
Keightly_script	1998	yes				yes	yes	yes	4.45	No	Keightley, 1998	NA	available from the corresponding author

– continued from previous page

Program Name	Year	u	M	NE	S	Windows	Mac	Linux	IF	GUI	Reference	DOI	Link
Lamarc	1999		yes	yes		yes	yes		4.221	No	Beerli & Felsenstein, 1999	NA	http://evolution.genetics.washington.edu/lamarc/index.html
Lamarc2	2006	yes	yes	yes		yes	yes	yes	4.894	NA	Kuhner, 2006	10.1093/bioinformatics/btk051	http://evolution.gs.washington.edu/lamarc/lamarc_prog.html
LDNe	2008			yes		yes	yes	yes	NA	Yes	Waples & Do, 2008	10.1111/j.1755-0998.2007.02061.x	implemented in NeEstimator v2
Lositan	2008				yes	yes	yes		3.781	NA	Antao et al., 2008	10.1186/1471-2105-9-323	https://github.com/tiagoantao/lositan
McDonald-Kreitman_online	2008				yes	yes	yes	yes	6.878	Yes	Egea et al., 2008	10.1093/nar/gkn337	mkt.uab.es/mkt
MCLEEPS	2000			yes			yes		4.687	No	Anderson et al., 2000	NA	https://www.stat.washington.edu/ thompson/Genepi/Mcleeps.shtml
mdiv	2001		yes			yes			4.803	No	Nielsen & Wakerly, 2001	10.1534/genetics.103.024182	https://bio.tools/mdiv
MEAdmix	2006			yes		yes			4.242	No	Wang, 2006	10.1534/genetics.105.054130	http://www.mybiosoftware.com/meadmix-1-0-molecular-estimator-admixture.html
MEGA6	2013				yes	yes	yes	yes	14.308	Yes	Tamura et al., 2013	10.1093/molbev/mst197	megasoftware.net/
MigEst	2014		yes			yes			6.494	NA	Wang, 2014	10.1111/mec.12806	http://www.zsl.org/science/software/migest
MIGRATE_N	2013	yes	yes			yes	yes	yes	4.087	No	Beerli et al., 2010	10.1534/genetics.109.112532	http://popgen.sc.fsu.edu/Migrate/Migrate-n.html
MIMAR	2007		yes	yes					5.169	No	Becquet & Przeworski, 2007	10.1046/j.1365-294X.2002.01650.x	http://przeworski.uchicago.edu/cbecquet/download.html
MISAT	1997			yes		yes			4.275	No	Nielsen, 1997	NA	https://www.mybiosoftware.com/misat-1-0-microsatellite-analysis-maximum-likelihood.html
MLNe	2001		yes	yes		yes			2.317	NA	Wang, 2001	10.1017/S0016672301005286	http://www.zsl.org/science/software/mlne
mlRho	2010	yes					yes	yes	6.457	No	Haubold et al., 2010	10.1111/j.1365-294X.2009.04482.x	http://guanine.evolbio.mpg.de/mlRho/
MSMC	2014			yes			yes	yes	29.352	No	Schiffels & Durbin, 2014	10.1038/ng.3015	https://github.com/stschiff/msmc
Nb_HetEx	2008			yes		yes			1.775	NA	Zhdanova & Pudovkin, 2008	10.1038/ng.3015	ftp://ftp.dvo.ru/pub/Personal/NB-Estimator
NeEstimator	2004			yes		yes	yes	yes	NA	Yes	Peel et al., 2004	NA	http://www.molecularfisherieslaboratory.com.au/neestimator-software/
NeEstimator2	2014			yes		yes	yes	yes	3.712	Yes	Do et al., 2014	10.1111/1755-0998.12157	http://www.molecularfisherieslaboratory.com.au/neestimator-software/
neighbor	2002		yes			yes			3.014	NA	Burczyk et al., 2002	10.1046/j.1365-294X.2002.01603.x	Upon request (from IJ Chybicki http://www.ukw.edu.pl/pracownicy/strona/igor_chybicki/english/)
NM+	2010		yes			yes			1.631	NA	Chybicki & Burczyk, 2010	10.1111/j.1755-0998.2010.02849.x	http://www.genetyka.ukw.edu.pl/ index_pliki/software.htm
nSl	2014				yes		yes		9.105	No	Ferrer-Admetlla et al., 2014	10.1093/molbev/msu077	http://www.nielsenlab.org/software/
Omegamap	2006				yes	yes	yes		4.242	No	Wilson & McVean, 2006	10.1534/genetics.105.044917	www.danielwilson.me.uk/omegaMap.html
OmegaPlus	2010				yes	yes		yes	5.323	No	Alachiotis et al., 2012	10.1093/bioinformatics/bts419	https://github.com/alachins/omegaplus

– continued from previous page

Program Name	Year	u	M	NE	S	Windows	Mac	Linux	IF	GUI	Reference	DOI	Link
ONeSamp	2008			yes		yes	yes	yes	NA	No	Tallmon et al., 2008	10.1111/j.1471-8286.2007.01997.x	currently not supported, version in preparation
OutFLANK	2015				yes	yes	yes	yes	3.148	No	Whitlock & Lotterhos, 2015	10.1086/682949	https://github.com/whitlock/OutFLANK
pcadapt	2017				yes	yes	yes	yes	7.059	No	Luu et al., 2017	10.1111/1755-0998.12592	https://CRAN.R-project.org/package=pcadapt
pegas	2010		yes			yes	yes	yes	4.877	No	Paradis 2010	10.1093/bioinformatics/btp696	https://cran.r-project.org/web/packages/pegas/index.html
Pool_hmm	2012				yes	yes	yes	yes	10.353	No	Boitard et al., 2012	10.1093/molbev/mss090	qgsp.jouy.inra.fr/
PopABC	2009	yes	yes	yes		yes	yes	yes	4.926	No	Lopes et al., 2009	10.1093/bioinformatics/btp487	https://code.google.com/p/popabc/
popgene	1997				yes	yes			0.231	Yes	Yeh & Boyle, 1997	NA	www.ualberta.ca/ fyeh/popgene.html
PSMC	2011			yes				yes	36.28	No	Li & Durbin, 2011	10.1038/nature10231	https://github.com/lh3/psmc
rehh	2012				yes	yes	yes	yes	5.468	No	Gautier & Vitalis, 2012	10.1093/bioinformatics/bts115	cran.r-project.org/web/packages/rehh/ index.html
rehh2	2017				yes	yes	yes	yes	7.059	No	Gautier et al., 2017	10.1111/1755-0998.12634	https://cran.r-project.org/web/packages/rehh/index.html
RpackageNB	2015			yes		yes	yes	yes	4.644	No	Hui & Burt, 2015	10.1534/genetics.115.174904	https://cran.r-project.org/web/packages/NB/index.html
SAMOVA	2002		yes			yes			3.014	NA	Dupanloup et al. 2002	10.1046/j.1365-294X.2002.01650.x	http://cmpg.unibe.ch/software/samova/
SelectionHapStats	2018				yes			yes	3.564	No	Harris et al., 2018	10.1534/genetics.118.301502	https://github.com/ngarud/ SelectionHapStats
Selecton	2007				yes	yes	yes	yes	6.954	No	Stern et al., 2007	10.1093/nar/gkm382	selecton.tau.ac.il/versions.html
SelEstim	2014				yes	yes	yes	yes	5.963	No	Vitalis et al., 2014	10.1534/genetics.113.152991	www1.montpellier.inra.fr/CBGP/software/selestim/
selscan	2014				yes	yes	yes	yes	9.105	No	Szpiech & Hernandez, 2014	10.1093/molbev/msu211	github.com/szpiech/selscan
SNeP	2015			yes		yes	yes	yes	NA	No	Barbato et al., 2015	10.3389/fgene.2015.00109	sourceforge.net/projects/snepnettrends
SPAM	2004		yes			yes			1.511	Yes	Debevec et al., 2000	NA	http://www.adfg.alaska.gov/index.cfm?adfg=fishinggeneconservationlab.software
spatpg	2016			yes	yes			yes	6.086	No	Gompert, 2016	10.1111/mec.13323	http://sourceforge.net/projects/spatpg/
Structure	2000		yes			yes	yes	yes	4.687	Yes	Pritchard et al., 2000	NA	https://web.stanford.edu/group/pritchardlab/structure.html
SweeD	2013				yes		yes	yes	14.308	No	Pavlidis et al., 2013	10.1093/molbev/mst112	https://sco.h-its.org/exelixis/web/software/sweed/
Sweep	2005				yes	yes	yes	yes	30.432		Sabeti et al., 2002	10.1038/nature01140	www.broad.mit.edu/mpg/sweep/
sweepfinder	2006				yes			yes	10.139	No	Nielsen et al., 2005	10.1101/gr.4252305	https://omictools.com/sweepfinder-tool
SweepFinder2	2016				yes		yes	yes	7.307	No	DeGiorgio et al., 2016	10.1093/bioinformatics/btw051	http://www.personal.psu.edu/mxd60/sf2.html
TempoFs	2007			yes		yes		yes	4.001	No	Jorde & Ryman, 2007	10.1534/genetics.107.075481	http://www.zoologi.su.se/ ryman/ TempoFs.zip

– continued from previous page

Program Name	Year	u	M	NE	S	Windows	Mac	Linux	IF	GUI	Reference	DOI	Link
Tess	2007		yes			yes	yes	yes	1.257	No	Chen et al., 2007	10.1111/j.1471-8286.2007.01769.x	http://membres-timc.imag.fr/Olivier.Francois/tess.html
ThetaCurve	2012	yes				yes	yes	yes	NA	No	n.a.	NA	http://www.plantsciences.ucdavis.edu/faculty/ross-ibarra/code/files/ThetaCurve.html
tm3	2002			yes		yes			4.483	NA	Berthier et al., 2002	NA	http://www.maths.bris.ac.uk/ mamab/software/
tmvp	2003			yes		yes			4.276	NA	Beaumont, 2003	NA	http://www.maths.bris.ac.uk/ mamab/software/
TreeSelect	2011				yes	yes	yes	yes	10.603	No	Bhatia et al., 2011	10.1016/j.ajhg.2011.07.025	hsph.harvard.edu/alkes-price/software
WFABC	2015		yes	yes	yes	yes	yes	yes	5.298	No	Foll et al., 2015	10.1111/1755-0998.12280	http://jjensenlab.org/wp-content/uploads/2016/02/WFABC_v1.1.zip
